# Remission of Type 2 Diabetes with Very Low-Calorie Diets—A Narrative Review

**DOI:** 10.3390/nu13062086

**Published:** 2021-06-18

**Authors:** Susan Juray, Kathleen V. Axen, Steven E. Trasino

**Affiliations:** 1 Nutrition Program, School of Urban Public Health, Hunter College, City University of New York, New York, NY 10035, USA; susan.juray@mountsinai.org; 2 Clinical Nutrition Department, Mount Sinai Hospital, New York, NY 10025, USA; 3 Department of Health and Nutrition Sciences, Brooklyn College, City University of New York, New York, NY 11201, USA; kaxen@brooklyn.cuny.edu

**Keywords:** type 2 diabetes, remission, very low calorie diets, hypocaloric diets

## Abstract

Very low-calorie diets (VLCD) are hypocaloric dietary regimens of approximately 400–800 kcal/day that result in 20–30% reductions in body weight, sometimes in just 12–16 weeks. A body of evidence demonstrates that adherence to VLCD in adults with type 2 diabetes (T2D) can result in marked improvements to glycemic control and even full T2D remission, challenging the convention that T2D is a lifelong disease. Although these data are promising, the majority of VLCD studies have focused on weight loss and not T2D remission as a primary endpoint. Moreover, there is a wide range of VLCD protocols and definitions of T2D remission used across these hypocaloric studies. Together the large degree of heterogeneity in VLCD studies, and how T2D remission is defined, leave many gaps in knowledge to efficacy and durability of VLCD approaches for T2D remission. This narrative review examines findings from a body of data from VLCD studies that specifically sought to investigate T2D remission, and discusses the efficacy of VLCD compared to other hypocaloric approaches, and who is likely to benefit from VLCD approaches for T2D remission.

## 1. Introduction

Type 2 diabetes (T2D) and its comorbidities have reached epidemic proportions globally [1], largely driven by high rates of obesity in adults and children. With less than 2% of cases of T2D entering spontaneous remission [2], the current clinical paradigm is that T2D is irreversible. Although current standard clinical care is effective in maintaining normoglycemia in T2D [3], long-term studies show that intensive glycemic control is not sufficient in halting T2D progression and decline in pancreatic β-cell function, or in mitigating the risk of cardiovascular disease [4,5]. Weight loss is the primary approach for nutritional management of T2D [6,7], and evidence shows that, compared to standard care, intensive multicomponent lifestyle interventions, including behavior change, physical activity and dietary energy restriction produce superior weight loss and improvement to glycemic control [8,9]. 

Dietary energy restriction approaches for management and treatment of T2D have largely focused on weight reduction through the use of either low-calorie diets (LCD) (1200–1500 kcal/day) [10,11,12], or very low-calorie diets (VLCD) (approximately 400–800 kcal/day) [13,14,15,16,17,18,19,20,21,22]. VLCD approaches have shown to be the most effective in producing rapid weight loss, improvement to pancreatic insulin secretory capacity, and reduction of hemoglobin A1c (HbA1c) to pre-diabetic and non-diabetic levels, sometimes within days [13,14,15,16,17,18,19,20,21,22,23,24,25]. However, despite this body of evidence [13,14,15,16,17,18,19,20,21,22,23,24,25], the long-term efficacy of VLCD for reversing T2D remains unclear, as many of these studies have been of short duration (< 12 weeks), did not have T2D remission as a primary endpoint, and varied greatly in their VLCD protocols, follow up duration, and definitions of T2D remission.

A recent body of evidence has demonstrated that with VLCD approaches, clinically defined and sustained (6–24 months) T2D remission can occur [17,20,21,22,23,24,25], raising the possibility for a cure of T2D. Evidence from two comprehensive randomized control trials (RCT) of VLCD [22,25], show that a VLCD intervention led to significant reductions in body weight and sustained T2D remission in 45–60% of subjects after 12 months, and in 35% of individuals at 24 months [22,25]. However, data also showed that approximately 30% of individuals with relatively longer duration T2D (3.8 years since diagnosis) did not achieve remission despite significant weight loss (i.e., non-responders) [26]. Also, consistent with numerous VLCD weight loss studies [27], approximately 25% of individuals who achieved remission from T2D, regained a significant proportion of their lost weight and had their T2D relapse at 24 months [23,26].

Collectively, these data demonstrate that it remains unclear what the efficacy and durability of T2D remission is, using VLCD approaches, and, equally important, a better understanding is needed of which individuals with T2D would most likely respond and benefit from VLCD approaches for T2D remission.

This narrative review will provide an overview of the studies which share similar VLCD approaches that specifically sought to measure T2D as a primary endpoint. This narrative review will also address who is likely to benefit, what the risks are, and how a VLCD approach compares to other less restrictive hypocaloric diets on remission of T2D.

## 2. Methods

Our objective was to compile a narrative overview of studies that specifically sought to examine whether a VLCD and solid food protocol results in durable remission of T2D. We compiled a list of intervention studies based on the following criteria: (i) used VLCD protocols based on the definition of a liquid diet formulation of approximately 400–800 kcal/day, (ii) included a solid food reintroduction phase, and (iii) specifically defined T2D remission in their methodology as a primary endpoint.

A search of the PubMed and EMBASE databases was conducted for English language original research articles and narrative reviews published from 2000 through January 2021 using the search terms “very-low calorie diet”, “very-low energy diet” and each of the following terms: “type 2 diabetes” “remission”, and “reversal”. A total of 202 results from this literature search were returned and then results were further evaluated for relevance and eligibility for inclusion in this review. Of the 202 articles, five (5) original articles met our inclusions of caloric range of VLCD, and for specifying remission of T2D as a primary endpoint. Table 1 summarizes the details and major findings of these 5 studies. 

## 3. Results and Discussion

### 3.1. Definition and Safety of Liquid Very Low-Calorie Diets (VLCD)

Historically, use of VLCDs for treatment of obesity gained considerable attention in the 1970s for their effectiveness in producing marked and rapid weight loss, often with body weight reductions of 20–30% in 12–16 weeks [30,31]. A VLCD protocol typically involves the replacement of all food with a medical grade liquid diet formulation that provides approximately 400–800 kcal/day for 12 to 16 weeks, followed by a structured solid food reintroduction phase with kcal/day tailored for weight loss maintenance [30]. Most VLCD liquid formulas consisted of approximately 50–60% of kcal from carbohydrate (maltodextrins and sucrose) to prevent ketosis, sufficient levels of essential fatty acids to meet the recommended dietary allowances, and high-biological-value protein (i.e., whey isolate) at 1.2–1.5 g/kg body weight to prevent loss of lean muscle mass during rapid weight loss [30]. Liquid VLCD formulas typically contained very little fiber and are sweetened with artificial sweeteners such as aspartame and sucralose [30]. Although VLCD formulas typically contain sufficient vitamins and minerals to meet the dietary requirements of adults, intake of a vitamin and mineral supplement is suggested [30], as an underestimation of micronutrient needs for obese adults, and those using VLCD has been reported [32,33].

Under medical supervision a VLCD is generally considered safe [30,31], but a number of side effects, particularly in the early stages of liquid diet administration, can occur, including fatigue, constipation, and diarrhea [30,31]. More serious adverse events are infrequent and include hyperuricemia, gout, and cardiac complications [30,31]. Due to lower fat intakes, concerns about development of gallstones with low calorie diets, including VLCD have been reported [30,34,35,36]. Replacing carbohydrate and protein with additional fat in liquid diet formulas could potentially promote gallbladder emptying which can reduce the risk of gallstone formation [30]. As unsupervised use of VLCDs can result in serious medical complications, intensive and comprehensive medical monitoring by a physician in conjunction with a registered dietitian (RD) is highly recommended [30,31].

Risk for hypoglycemia can increase with combined use oral hypoglycemic agents, in particular sulfonylureas (i.e., gliclazide, glimepiride), or exogenous insulin, with a VLCD protocol [31]. Therefore, close medical supervision and dose adjustments or discontinuation of oral hypoglycemic agents with a VLCD is recommended, particularly during the total food replacement phase [31]. Moreover, individuals should be trained in the use of home blood glucose monitoring during any adjustment period of antidiabetic medications [31]. Medications for hypertension are also often decreased or discontinued at the onset of a VLCD protocol, but should be re-introduced if hypertension is elevated or returns [31]. Also, with the danger of hypotension and electrolyte disturbances, discontinuation of diuretics should also be considered [31].

### 3.2. Definition of Type 2 Diabetes (T2D) Remission

The specific use of VLCD as a protocol for T2D remission is recent and relatively unexplored, as the majority of VLCD studies to date have focused on weight loss [27,30,37,38]. Although there have been numerous studies demonstrating various degrees of improvements to glycemic control with VLCD [13,14,15,16,18,19], the majority of these have not specifically defined the parameters of T2D remission as a primary endpoint. 

In response to growing evidence of T2D remission after bariatric obesity surgery (reviewed in [39]), in 2009, Buse et al. [40] provided the following working definition of T2D remission: “Remission is defined as achieving glycemia below the diabetic range in the absence of active pharmacologic (antihyperglycemic medications, immunosuppressive medications) or surgical (ongoing procedures such as repeated replacements of endoluminal devices) therapy. Remission can be characterized as partial or complete: (1) partial remission is sub-diabetic hyperglycemia (A1c not diagnostic of diabetes [<6.5%, <48 mmol/mol], fasting glucose 100 to 125 mg/dL) and (2) complete remission is a return to “normal,” measures of glucose metabolism (A1c in the normal range [<5.7%, 39 mmol/mol], fasting glucose <100 mg/dL). Partial or complete remission requires at least 12 months of the absence of active pharmacologic therapy or ongoing procedures to be characterized in a remissive state.”

A recent meta-analyis shows that the large degree of heterogeneity in how T2D remission is defined remains a major barrier to arriving at consensus on the efficacy of VLCD and T2D remission [41]. With the exception of Lim et al. [17], all the studies included in this narrative review use components of the Buse et. al. [40] definition of T2D remission.

### 3.3. Effect of Very Low-Calorie Diets on Remission of T2D

We summarized five (5) VLCD studies that specifically examined VLCD for T2D remission, and defined T2D remission parameters in their methods [17,20,21,22,23,24,25] (Table 1). The Counterpoint study (Counteracting Pancreatic Inhibition by Triglyceride) Lim et al. [17], was among the first studies to comprehensively explore T2D remission with VLCD. More specifically, it sought to examine the relationship between excess pancreatic lipid and impaired insulin responses in the reversal of T2D, and to test the twin cycle hypothesis for the onset of T2D (reviewed in [42]). The Twin Cycle hypothesis purports that in obesity and pre-diabetes, fatty liver and hepatic overproduction of triglyceride-rich very-low density lipoprotein (VLDL) has a spillover effect, leading to ectopic pancreatic fat accumulation, impaired β-cell function and onset of T2D [42]. The Counterpoint study used an 8-week VLCD protocol of 600 kcal/day (25% of energy requirement) in 11 obese subjects with T2D who had relatively short-duration disease of less than 4 years since diagnosis [17].

All metabolic parameters were compared to baseline recruitment values and against those of age and sex-matched non-diabetic controls [17]. They reported that, after 7 days of the VLCD, participants had reductions in fasting plasma glucose (FPG) to non-diabetic levels (<100 mg/dL), as well as significant reductions in fasting plasma insulin (*p* < 0.05), and triglyceride (*p* < 0.01). The normalization of FPG to non-diabetic levels after just 7 days occurred with a modest weight loss of approximately 4% of initial body weight, but a 30% decrease in ectopic pancreatic and hepatic triglyceride [17]. At the completion of 8-weeks, the VLCD intervention resulted in a body weight reduction of 15 kg, and all subjects maintained the 30% reductions in intrahepatic and pancreatic lipid levels, with 73% of subjects maintaining non-diabetic FPG levels [17]. The Counterpoint study provided proof of concept that with VLCD normalization of glycemic control in T2D is possible, and evidence that short-term VLCD promotes marked improvement in hepatic insulin resistance and normalization of β-cell function [17].

Given the positive results of the Counterpoint study in participants with relatively new onset of T2D [17], a larger and longer follow-up study by Steven et al. [21] sought to examine the effects VLCD in subjects with relatively longer T2D disease duration. Coined the Counterbalance study (Counteracting BetA cell failure by Long term Action to Normalize Calorie intakE), this study expanded the VLCD protocol from Lim et al. in three phases: 8 weeks of VLCD liquid formula (624–700 kcal/day), followed by 2 weeks of an isocaloric solid food diet, and then by a structured and individualized 6-month weight maintenance program. Subjects that achieved non-diabetic T2D remission were categorized as “responders”, defined by achievement of a FPG of <125 mg/dL and HbA1c levels of <6.5% (<48 mmol/mol) at the 2-month and 6-month follow-up periods. They found, similar to Lim et al. [17], that in some individuals FPG levels were rapidly reduced to non-diabetic levels within 7 days after the onset of the VLCD [21]. After 8-weeks on the VLCD, mean weight loss was approximately 15% of baseline body weight among both responders and non-responders, which then remained unchanged in both groups over the 6- month duration of the study. However, after 8 weeks of VLCD, 87% of subjects with relatively shorter T2D duration (<4 years since diagnosis) had non-diabetic FPG levels, whereas just 50% of the long- duration T2D group (8–23 years since diagnosis) achieved non-diabetic FPG. At a 6-month follow up, during the weight maintenance phase, 40% of the participants in the VLCD intervention arm were classified as responders and in T2D remission [21]. Compared to non-responders, 60% of responders had relatively shorter duration disease of <4 years at baseline, lower FPG, HbA1c, and higher first phase insulin responses [21]. Also, consistent with findings in Lim et al. [17], improvements to β-cell function in responders occurred with concomitant reduction in pancreatic fat levels [21]. The 10-year Q-RISK score, a metric used to assess cardiovascular disease (CVD) risk [43], was reduced from 23% to 7% in responders from the Counterbalance study, which is consistent with a large body of data demonstrating significant reductions in CVD with weight loss and glycemic control in T2D [44,45]. 

Similarly, a small prospective VLCD study by Umphonsathien et al. [24], specifically examined T2D remission in 19 overweight adults (mean body mass index [BMI] 27.7), with an average T2D duration of 2 years (range 0.4–8 years). Subjects were administered a VLCD of 600 kcal/day for 10 weeks, followed by a 4-week gradual solid food reintroduction phase, and an increase in energy intake up to 1500 kcal/day. Dietary compliance was measured using a daily food record and urine ketone levels. They defined T2D remission as FPG level <126 mg/dL, HbA1c <6.5% (<48 mmol/mol), and the discontinuation of all antidiabetic medications. Dietary compliance was reported at 90%, and they reported that at weeks 4, 8 and 12 of the re-feeding phase, approximately 40%, 75% and 70% of subjects were in T2D remission, respectively [24]. Over 12 weeks, those who achieved remission had a mean weight loss of 9.5 kg or 12% of initial body weight. This small study found, similar to the Counterpoint and Counterbalance studies [17,20,21], that improvement in glycemic control was apparent in the first 7 days of onset of the VLCD and that duration of T2D was a predictor of remission (mean duration of 2 years vs. 6 years in non-responders) [24]. While it would have been informative, rates of remission were not reported after the re-feeding period. Although this study, along with Counterpoint and Counterbalance studies, provides proof of concept that VLCD can result in T2D remission with a high response rate, these studies lack the appropriate controls, and had relatively short follow-up of 2-6 months. 

The Diabetes Remission Clinical Trial (DiRECT) sought to address these limitations with a rigorous and comprehensive study of a VLCD approach for T2D remission that spanned 24 months [22,23]. DiRECT recruited 306 overweight and obese adults aged 20–65 with a BMI range of 27–45 kg/m, diagnosed with T2D within the past 6 years [22]. Exclusion criteria included diagnosis of very advanced T2D, defined as requiring exogenous insulin or with an HbA1c of 12% (108 mmol/mol) or greater [22]. All subjects were then cluster randomized to either a VLCD protocol, or a standard diabetes care intervention [22]. The VLCD intervention arm involved 3 phases: 1) Total diet replacement phase: a daily liquid formula diet replacement providing 825–853 kcal/day for 3–5 months (duration based on individualized weight loss goals); followed by 2) Food reintroduction phase: a stepped food reintroduction and increase in daily kcal for 6–8 weeks, adjusting individualized caloric intake for maintaining weight loss; and 3) Weight loss maintenance phase: a 24-month, multi-pronged, individualized medical and behavioral support model called the Counterweight-Plus program delivered either by a nurse or registered dietician for long-term weight maintenance [22,23]. The Counterweight-Plus program has been shown to be effective in real-life primary care settings in the community and, because it can be administered either by dietitians or a nurse, it reduced cost VLCD treatment to less than half of the average cost for treating an individual with T2D in the U.K. [28]. Remission from T2D was defined as HbA1c < 6.5% (<48 mmol/mol) after at least 2 months without the need for antidiabetic medications. The attrition rate for the DiRECT intervention group was 25% at 12 months [22], which is superior to the 25–50% dropout rates in the first 3 to 6 months of prior VLCD studies [27,46,47]. Results from the primary outcomes of T2D remission and weight loss were reported based on an intent-to-treat model [22,23].

At 12 months, within the VLCD intervention arm, 46% of participants achieved remission of T2D, mean weight loss was 10 kg, and 25% lost over 15 kg of body weight [22]. Remission rates and weight loss in the standard care arm at 12 months were 4% and 0% respectively [22]. Almost 74% of subjects in the VLCD intervention arm no longer needed antidiabetic medications compared to 18% in the standard care arm at month 12 of the weight maintenance phase [22]. Subjects in the VLCD intervention arm also had marked reductions in blood pressure, with 48% of participants no longer requiring the use of antihypertensive drugs, compared to no reductions in antihypertensive drug use in the standard care arm [22]. A 24 month follow up showed that 35.6% of subjects in the VLCD intervention arm remained in T2D remission, and among those who achieved remission, 70% maintained a weight reduction of over 15 kg [23]. Improvement in quality of life and general well-being were higher in subjects in the VLCD intervention than those in the standard care arm [23]. 

The Diabetes Intervention Accentuating Diet and Enhancing Metabolism (DIADEM-I) trial is another RCT in similar size and scope to the DiRECT that also specifically sought to examine T2D remission as a primary endpoint [25]. DIADEM-I recruited 147 overweight adults aged 18–50 with a BMI 27 kg/m2 or greater, and a mean T2D duration of <3 years (mean of 21.2 months) [25]. DIADEM-I defined T2D remission using a similar parameter as DiRECT, of HbA1c < 6.5% (<48 mmol/mol), and being free of antidiabetic medications for at least 3 months [25]. However, unlike DiRECT, DIADEM-I also included a more rigorous level of remission termed “normoglycemia” defined as HbA1c <5.7% (<39 mmol/mol), with no antidiabetic medications for at least 3 months [25]. All participants stopped use of antidiabetic medications, and were then randomized to receive either a supervised intervention of 12 weeks of a either a total diet replacement of a VLCD liquid formula of 800–820 kcal/day, followed by a structured 12-week food reintroduction phase (n = 70), or standard care (n = 77), based on American Diabetes Associations (ADA) guidelines [48]. Subjects in the VLCD arm also received behavioral support using a multi-component approach called the Specialist Lifestyle Management (SLiM) weight loss program, that was reported to be successful in facilitating weight loss in obesity [29]. The follow up period was 12 months from baseline. Consistent with the DiRECT [22], subjects in the VLCD intervention arm of DIADEM-I had a mean weight reduction of 11.98 kg at month 12, with 15% of subjects achieving a weight loss of greater than 15% of body weight, compared to 3.98 kg of weight loss in the control arm [25]. T2D remission and normoglycemia occurred in 61% and 33% of participants respectively in the VLCD intervention arm, and 12% and 4% of participants in the control arm. Similar to the DiRECT [22], the drop-out rate in the VLCD arm was 18.9%, (15/79), and in the control arm 12.6% (10/77) [25].

### 3.4. Comparison between a Very Low-Calorie Diet and a Low-Calorie Diet for T2D Remission

The DiRECT and the DIADEM-I are the only large-scale RCTs of VLCD to examine T2D remission as a primary endpoint. However, it is appropriate to compare their findings to those of the Look AHEAD study [10,49], the largest-RCT to examine the potential of an intensive lifestyle intervention (ILI) of diet, exercise and behavioral support to achieve and maintain long-term weight loss among obese individuals with T2D [10,49]. LookAHEAD randomized 2241 participants to an ILI of a low-calorie diet of 1200–1500 kcal/day, physical activity and intensive behavioral support [10,49]. The ILI group was compared to individuals in a standard care of diabetes support and education intervention (DSE). At the 4-year outcomes assessment, approximately 50% of the ILI group maintained the loss of 5% of their baseline body weight, and 27% lost and maintained 10% of their baseline body weight [10,49]. Although T2D remission was not a primary endpoint, using a definition of FPG of <126 mg/dL, HbA1c of <6.5% (<48 mmol/mol), without requiring any antihyperglycemic medication, post-hoc analysis in the Look AHEAD reported that 11.5% participants in the ILI group had partial or complete remission of T2D by 12 months, and 9.2% had T2D remission at 24 months, compared to 2% remission in the standard care group [10]. Also, individuals that achieved T2D remission in the ILI group, maintained 6.3% of their lost weight, compared to 0.9% weight loss maintenance in the standard care group [10,49]. A follow-up showed a decline in T2D remission rates, with 7.5% of ILI participants in remission after 4 years [50]. Compared to the LookAHEAD, mean weight loss (10 kg vs. 8.6 kg) was similar, but T2D remission rates at 12 months (46% vs. 9.2%), were superior in the DiRECT [10,22]. It is unclear why, despite having less than 15% difference in the mean weight loss reductions after 12 months, that the differences in T2D remission rate in the DiRECT study was markedly greater (>130%) than that of LookAHEAD.

A study by Sarathi et. al. [51] sought to examine T2D remission, but with more moderate calorie restriction of 1500 kcal/day than those typical of VLCD. They recruited 28 adults (mean 24 years of age) with newly diagnosed T2D (<12 months), who were then counseled to consume 1500 kcal/day using solid foods and to undertake daily brisk walking for 1 hour per day with follow-ups at 3, 12 and 24 months. After 3, 12 and 24 months, remission, defined as FPG of <100 mg/dL and HbA1c of <5.7% (< 39 mmol/mol), was reported in 53.1%, 50% and 46.9% of subjects [51]. Mean weight loss at 3, 12 and 24 months was 4.97 kg, 6.56 kg and 7.66 kg, respectively. The 24-month mean was 8.2% of initial body weight, which is similar to the 7.6% weight reduction reported in the VLCD arm in the DiRECT at 24 months [23]. 

Lastly, a small study (n = 8), by Petersen at al. [52], demonstrated in obese subjects with T2D, that euglycemia and improved insulin sensitivity can be achieved with a modest energy restriction of ~1200 kcal/day for 7 weeks [50]. The authors noted that normalization of T2D parameters occurred in all 8 individuals, and with a body weight reduction of 8 kg, but an 80% reduction of intra-hepatic lipids [52]. These data suggest that T2D remission, albeit at lower rates than observed with VLCD [17,20,21,22,23,24], is possible with even modest calorie restriction and exercise. This opens the possibility that individuals who struggle with the more aggressive calorie restriction of VLCD have alternatives for T2D remission approaches.

### 3.5. Characteristics of Good Candidates for Use of VLCD for T2D Remission

Although weight loss was among the strongest predictors of T2D remission [22,53], evidence from the Counterbalance and DiRECT studies demonstrated that approximately 30–56% of individuals that lost appreciable body weight (more than 15 kg) still did not achieve T2D remission (non-responders) [21,26], demonstrating that weight loss in itself is not sufficient for reversal of T2D. A post-hoc analysis in a subset of the DiRECT cohort [54], found that non-responders had significantly longer duration of T2D since diagnosis (3.8 vs. 2.7 years (responders), *p* = 0.02). It was also reported that compared to responders, non-responders at 24 months had higher pancreatic fat, and poorer β-cell function, with approximately 50% lower first phase insulin secretory response [54]. No baseline demographic or metabolic parameters, including age, sex, BMI, liver or pancreatic fat content, differed between non-responders and responders [54]. Similarly, the Counterbalance study [21] found a significant gap in T2D duration between VLCD responders and non-responders (3.8 vs. 9.8 years), and also found age to be a factor, with non-responders being ~8 years older than responders (52 vs. 59.9 years). Furthermore, the DIADEM-I study [25], which recruited adults with relatively short duration T2D of 3 years or less, reported a higher percentage of remission at 12 months (60%) compared to the DiRECT (46%), which recruited adults with a mean T2D duration of 6 years or less [22], supporting a possible role of disease duration in predicting T2D remission. The LookAHEAD study also reported that T2D remission was highest among subjects with the shortest length of T2D (≤2 yrs.) [10]. Furthermore, several bariatric obesity surgery studies have shown that baseline pancreatic β-cell function is a strong predictor of T2D remission [55,56,57].

Together these data suggest that only individuals with a relatively shorter duration disease, and a critical mass of pancreatic β-cells and/or β-cell function, should be candidates for hypocaloric T2D remission protocols. However, surprisingly, post-hoc analysis of the original full DiRECT cohort found that weight loss, not T2D duration, was the strongest predictor of T2D remission [53]. Also, though the DIADEM-I reported higher rates of remission at 12 months compared to the DiRECT, the VLCD participants in the DIADEM-I were appreciably younger than subjects in DiRECT VLCD arm (41.9 vs. 52.9 yrs.). Participants that achieved T2D remission in the Counterbalance study were also younger than participants that did not (52.0 vs. 59.9 yrs.) [21], together suggesting that age itself may be a key factor in determining an individual’s potential for T2D remission.

A recent study reported that higher levels of the orexigenic hormone ghrelin during the liquid diet phase of VLCD is a predictor of weight regain [58], suggesting that pre-enrollment ghrelin levels may be an effective screening tool. Evidence suggesting that disease duration and β-cell function are predictors of T2D remission is consistent with the pathophysiology of T2D, in which the onset of pancreatic β-cell loss and secretory insufficiency represent a key clinical transition from insulin resistance to T2D [59]. Nevertheless, until there is a better understanding of the relationship between disease duration, pancreatic β-cell function, and potential for T2D remission, it is currently not possible to screen candidates most likely to succeed in achieving T2D remission. This is particularly important knowing the challenges, potential health risks and costs that are associated with undertaking a medically supervised VLCD protocol [31].

### 3.6. Degree of Weight Loss Necessary for T2D Remission

The current arguments against the use of a VLCD for weight loss are based on a body of data demonstrating that VLCD approaches are only effective in producing rapid weight loss in the short-term (<12 months), and that after the reintroduction of solid food in the long-term (≥24 months), marked weight regain is typical [27,31,45]. Data from a number of meta-analyses of VLCDs are in agreement that in the long-term (1–5 years of follow up), most individuals fail to maintain the 15–20% weight reductions that are typical of VLCDs [27,28,45]. However, data show that various degrees of T2D remission can occur across a wide range of weight loss, sometimes before any appreciable reduction in body weight occurs [10,15,18,20,23]. For example, a weight loss VLCD study by Henry et. al. [15] of 30 obese adults (mean BMI >35) with T2D, reported that within just 10 days of the VLCD, 87% of intervention subjects (26/30) showed significant reductions (*p* < 0.001) in FPG, insulin levels, and hepatic glucose output (HGO), while losing only ~4% of their initial body weight [15]. In the DiRECT, at 24 months T2D remission in the VLCD arm was reported in 5% of individuals that lost as little as 5 kg of weight, almost 30% of individuals that lost 5–10 kg of weight, and almost 60% of participants that lost 10–15 kg of weight, or 10–15% of their initial body weight [23]. No data on remission within ranges of weight loss was reported in the DIADEM-I study [25]. Evidence that more modest weight loss and T2D remission was also reported in the LookAHEAD study [10], which showed a significant *(p* < 0.01) association between T2D remission across all tertiles of weight loss in the ILI group [10].

Together, these data show that with VLCD, short-term onset of T2D remission can occur with even negligible weight loss, and for some individuals, modest reductions in body weight of 10–15% that have been documented in numerous VLCD studies [27,38], are sufficient for maintaining T2D remission in the long-term. These data also suggest that, unlike typical anti-obesity approaches that emphasize maximum weight loss [27,46], future VLCD practices for T2D remission should favor highly flexible and tailor-made weight loss targets that consider individual responses.

### 3.7. Comparison of VLCD Approaches with Bariatric Surgery for T2D Remission

A body of data shows that morbidly obese subjects with T2D that undergo bariatric surgery can achieve, sometimes within days, non-diabetic FPG glucose levels [39]. A number of side-by side studies comparing the magnitude of short-term normalization of T2D parameters between VLCD and gastric bypass surgery, show that - both VLCD and bariatric surgery produce similar improvements to FPG and HbA1c [60,61,62,63,64], and marked calorie restriction itself, not weight loss, is a key contributor to T2D remission in the short- term (<4 weeks). To date, just one study the examined the side-by-side improvements to T2D glycemic control between VLCD and the most common bariatric surgery, Roux-en-Y Gastric Bypass (RYGB), in the medium-term (3 months) [65]. That study showed that after 3 months, FPG, HbA1c and HOMA-IR were similarly reduced by VLCD and RYGB, though weight loss was superior with RYGB [65].

Although long-term T2D remission with bariatric surgery varies greatly depending on the type of surgery, and other factors such as severity of baseline T2D [39], remission rates between 30–63% over a range of 1–5 year follow ups have been reported with bariatric surgery [39,66,67]. For example, a large meta-analysis by Arterburn et al. [66], showed that at 1, 3, and 5 years after RYGB bariatric surgery, 37.1%, 63.3%, and 68.2% of individuals achieved complete T2D remission (defined as discontinuation of T2D medication, FPG <100 and/or HbA1c levels <6.5% (<47.5 mmol/mol) for ≥ 90 days [66]. Complete T2D remission after 2 years was reported in approximately 55% of subjects [66]. Another meta-analysis found that for all forms of bariatric surgeries (i.e. gastric bypass, sleeve gastrectomy, and gastric banding), complete T2D remission was achieved by 66.6% of subjects between 2–5 years post-operatively [67].

Using a similar definition of T2D remission, data from the DiRECT shows that after 1 and 2 years, remission was reported in 45.6% and 35.6% of subjects [23]. These data suggest that VLCD and bariatric surgery produce similar reductions to T2D parameters in the short- to medium-term, but in the long-term (>2 years), remission rates are superior with bariatric surgery. However, higher T2D remission was reported at 1 year in DIADEM-I compared to those reported by Arterburn et al. (60% vs. 37.1%) [66]. Subjects in the DIADEM-I were on average younger (41.9 vs. 49.6 years), and with shorter duration T2D (<3 vs. 4.5 years) than the bariatric surgery subjects examined by Arterburn et al. [66], suggesting that for shorter duration T2D, VLCD may be as good or better than bariatric surgery in producing T2D remission. However only with longer follow up studies (> 2 years) of VLCD on T2D remission can a reliable comparison be made between these two T2D treatment approaches.

Disadvantages of bariatric surgery include the risk of surgical and post-surgical complications, such as wound infection and thromboembolism [68,69], and significant financial burden, with a mean cost of $14,389 in the U.S. [70]. Although some adverse health risks are associated with use of VLCD [30] (see above), data from the DiRECT suggest that VLCD can be administered with few health risks [22]. Compared to previous VLCD studies [28,29,32,33], the DiRECT study reported a smaller number of adverse events, including gallstones, which were 7%, (11/157) in the VLCD arm, and 13% (19/149) in the standard care arm [22]. No differences in adverse events between VLCD and control arms was reported in the DIADEM-I [25]. Therefore, given the evidence that VLCD approaches are effective in achieving clinically meaningful T2D remission [22,23,25], and are comparably safer than invasive surgery, a VLCD could represent a viable alternative to bariatric surgery for individuals with the goal of achieving T2D remission.

## 4. Summary and Conclusions

In this review we examine the findings from five (5) studies that specifically sought to investigate the effect of VLCD on remission of T2D [17,20,21,22,23,24,25]. A limitation of this narrative review is the absence of a quantitative treatment of this topic, i.e., meta-analysis. However, within the scope of the five (5) intervention trials reviewed here, including a critical analysis of the DiRECT cohort, the largest RCT to examine VLCD in T2D, data show that a VLCD approach results T2D remission in approximately 50% of subjects after 12 months, and 30% of subjects after 24 months [22,23,25]. Data from DiRECT and a number of these VLCD studies also show that subjects with a relatively shorter duration of T2D and better pancreatic β-cell function, are more likely to achieve T2D remission [10,15,21,54]. 

However, these findings provide little by way of developing specific and effective screening criteria for VLCD T2D remission protocol candidates, as there is a large degree of heterogeneity in the definition of short and longer duration T2D across these studies [21,23,54]. As it stands, evidence suggests that only subjects with a relatively recent T2D diagnosis may benefit from VLCD or other energy restriction approaches to elicit T2D remission. Nevertheless, there is a need for more specific biomarkers and screening methods in order to determine those most likely to benefit from a VLCD T2D remission approach. 

Currently, VLCDs are typically reserved for individuals who are severely and morbidly obese (BMI >30–35), who are resistant to standard treatments, and require medically justified rapid weight loss, such as prior to orthopedic surgery or organ transplant [30,71]. A VLCD is also commonly used 2–3 weeks prior to bariatric surgery to reduce visceral and liver fat mass, thereby minimizing peri- and post-surgical complications [72]. Nevertheless, due to cost, and questions of long-term efficacy and potential health risks, including fatigue, diarrhea, and gall stones [30,34,35], VLCD approaches remain outside of standards of clinical care for the treatment of obesity and T2D [31]. The recent endorsement by the American Diabetes Association for short-term usage of VLCDs for improving glycemic control is noteworthy [7], and evidence that VLCD is effective in reversing T2D in adolescents [73], suggests that more medical bodies may reconsider their positions on the use of VLCD in the treatment of T2D. As such, in the U.K., the National Health Service (NHS), recently announced the use of VLCD for remission treatment of T2D in some regional locations based on the DiRECT Counterweight program [28,74]. The NHS- sponsored implementation of VLCD protocols for treatment of T2D will be an invaluable test balloon, not only for the efficacy of VLCDs, but also on the primary care-based model for delivering an intensive diet and lifestyle approach for T2D remission. Such approaches hold the potential to provide a viable dietary, non-pharmacological approach for reversing T2D, as well as to mitigate the risk for T2D-related comorbidities, including heart disease, renal disease, and retinopathy. Although these studies have provided insight into the real potential for reversibility of T2D with weight loss after extreme caloric restriction, they are limited by their lack of long-term follow-up. A critical question also not addressed in these studies, is whether self-directed and long-term adherence to VLCD protocols are feasible, effective, and safe. There are limited data supporting long term maintenance of weight loss and subsequent glycemic improvements in response to VLCDs. Although evidence suggests that with intensive behavioral support, durability of weight loss with VLCD is improved [27,75,76,77], successful long-term adherence to these dietary approaches will continue to be challenging among individuals who lack optimal support for sustained behavior change. Until further research on long-term sustainability of VLCD is conducted, approaches to meal planning should be individualized, whereby physicians and RDs provide one-on-one guidance and support along the way.

## Figures and Tables

**Table 1:** Very low-calorie diet (VLCD) interventions for remission of type 2 diabetes (T2D).

Study Reference	Study Objective and Design	Follow Up Period	Participants	VLCD Intervention	Control	Counseling and Behavioral Support:	Measure of Dietary Adherence	VLCD Drop-Out Rate	Definition of Remission	Mean Weight Loss (% of Initial Body Weight)	Remission (%)
Lim et al., 2011 [17]	Counterpoint study -examined VLCD on β-cell function and liver insulin sensitivity in short duration type 2 diabetes. Design: Single arm case control intervention study	2 months	24 adults (35–65 years, BMI 25–45 kg/m^2^) with type 2 diabetes (T2D) of <4 years since diagnosis (HbA1c 6.5% to 9.0%). All antidiabetic medications discontinued at study onset.	8 weeks of a liquid meal replacement formula (46.4% carbohydrate, 32.5% protein, and 20.1% fat; vitamins, minerals, and trace elements (510 kcal/day) Optifast; Nestlé Nutrition, Croydon, UK), and 3 daily portions of nonstarchy vegetables for total daily caloric intake of 600 kcal. Participants were also directed to drink at least 2 L of calorie-free beverages/day and maintain their typical level of physical activity.	9 non-diabetic controls matched for weight, age and sex.	After initial nutrition counselling, ongoing diet adherence support was provided by telephone.	Adherence was determined using blood ketone levels	27.0%	Fasting plasma glucose levels (<126 mg/dL), hepatic insulin sensitivity determined by suppression of hepatic glucose production, and normalization of β-cell function (determined by fasting insulin secretion rate)	After 8 weeks of VLCD: 15% ( ± 1%) of initial body weight	After 8 weeks of VLCD: 100% (24/24)
Steven et al., 2015 [20], 2016 [21]	Counterbalance study (Counteracting Beta cell failure by Long term Action to Normalize Calorie intakE) -examined VLCD on short duration and long duration type 2 diabetes. Design: Single-arm intervention study	2 and 6 months	30 adults (25–80 years, BMI 27–45 kg/m^2^), T2D of <4 years (HbA1c 7.2%) to >8 years (HbA1c 8.6%) since diagnosis. All antidiabetic medications discontinued at study onset.	8 weeks of liquid meal replacement formula (43% carbohydrate, 34% protein and 19.5% fat; vitamins, minerals and trace elements; (624 kcal/day); Optifast; Nestle Nutrition, Croydon, UK), and up to 240 g of nonstarchy vegetables per day for a total daily energy of 624–700 kcal. Followed by a stepped food reintroduction phase with gradual reintroduction of solid food over 7 days. Participants were also directed to drink at least 2 L of calorie-free beverages/day and maintain their typical level of physical activity.	None	After initial nutrition counselling, ongoing diet adherence support provided through telephone, e-mail, text, or face-to-face contact.	Adherence to liquid diet was determined using blood ketone levels and to isocaloric solid food intakes from resting energy expenditure measured by indirect calorimetry.	4.0%	Fasting plasma glucose levels (<126 mg/dL), and achieving nondiabetic HbA1c levels (<6.5%)	After 2 months of refeeding: 14.8% (±0.8%) and 14.4% (±0.7%) of initial body weight in short- and long-T2D duration groups respectively. After 6 months of refeeding: 15.8% (±0.5%) and 13.6% (±0.7%) of initial body weight in short- and long-T2D duration groups respectively.	After 2 months of refeeding: 69% (20/29) After 6 months of refeeding: 43% (13/30)
Lean et.al., 2018 [22], 2019 [23]	Diabetes Remission Clinical Trial (DiRECT). First comprehensive study of VLCD for T2D remission in adults Design: Cluster Randomized Control Trial	12 and 24 months	306 adults (20–65 years, BMI 27–45 kg/m^2^) with T2D of ≤6 years since diagnosis (HbA1c > 6%). All antidiabetic medications discontinued at study onset.	16–20 weeks of total liquid meal replacement formula (825–853 kcal/day (59% carbohydrate, 13% fat, 26% protein, 2% fiber), followed by a structured food reintroduction of 2–8 weeks (50% carbohydrate, 35% fat, and 15% protein). All subjects encourage to maintain usual physical activity during meal replacement stage; and step counters were provided during food reintroduction stage with a target of up to 15, 000 steps per day.	Standard-care based on National Institute of Health and Care Excellence in England and the Scottish Intercollegiate Guidelines Network in Scotland.	Counterweight-Plus weight management program [28]. Individualized behavioral support for maintaining at least 15 kg weight loss, but with flexible weight loss goals based on individualized needs.	Not specified	25.0%	HbA1c < 6.5% after at least 2 months off all antidiabetic medications after at least 2 months after month 12.	After 12 months of refeeding: 10.0% (±8%) and 1.0% (± 3.8%) of initial body weight in the VLCD and controls arms respectively. After 24 months of refeeding: 7.5% (±6.4%) and 2.3% (± 5.2%) of initial body weight in the VLCD and controls arms respectively.	After 12 months of refeeding: 46% (68/149) and 4% (6/149) T2D remission in the VLCD and control arms respectively. After 24 months of refeeding: 36% (53/149) and 3% (5/149) T2D remission in the VLCD and control arms respectively.
Umphonsathien, et. al 2019 [24]	Single-arm intervention study	14 weeks	20 adults (20–60 years, BMI of 23–30 kg/m^2^), T2D of <10 years. All antidiabetic medications discontinued at study onset.	10 weeks of 600 kcal/day followed by a step-wise increased in kcal/day at week 10 (800 kcal/ day), week 11, (1000 kcal/day), and week 12 (1200 kcal/day), week 13 (1500 kcal/day). Participants were directed to maintain their typical daily lifestyle throughout the study.	None	After initial nutrition counselling, ongoing diet adherence support provided through telephone.	Weekly dietary recall and weekly urine ketone levels measurement to measure dietary compliance.	10.0%	Fasting plasma glucose levels (<126 mg/dL), HbA1c levels (<6.5%), and no longer needing antidiabetic medications.	After week 14 of refeeding: 13.3% (± 2.2%) of initial body weight	After week 14 of refeeding: 79% (15/19).
Taheri, S., et al. 2020 [25]	The Diabetes Intervention Accentuating Diet and Enhancing Metabolism (DIADEM-I). Design: Open-label, parallel-group, randomized controlled trial.	12 months	158 adults (18–50 years, BMI ≥ 27 kg/m^2^) with type 2 diabetes of ≤3 years since diagnosis. All antidiabetic medications discontinued at study onset.	12 weeks of total liquid meal replacement formula (800–820 kcal/ day (57% carbohydrate, 14% fat, 26% protein, 3% fiber), followed by a structured food reintroduction of 12 weeks. All subjects encourage to maintain usual physical activity during meal replacement stage; and step counters were provided during food reintroduction stage with a target of up to 10, 000 steps per day and to increase activity to at least 150 min per week.	Standard care based on the American Diabetes Associations (ADA) guidelines.	Specialist Lifestyle Management (SLiM) weight loss program [29]. A structured education and self-management weight management program.	Not specified	18.9%	Two levels of remission. (1) T2D remission: HbA1c < 6.5% and free of antidiabetic medications for at least 3 months. (2) Normoglycemia: HbA1c < 5.7% and no antidiabetic medications for at least 3 months.	After 12 months of refeeding: 11.9% (±9.4%) and 3.9.0% (± 5.2%) of initial body weight in the VLCD and controls arms respectively.	After 12 months of refeeding: (1) T2D remission: 60% (42/70) and 12% (9/77) in VLCD and controls arms respectively. (2) Normoglycemia: 33% (23/70) and 4% (3/77) in VLCD and control arms respectively.
